# Supplementary material for: Cardiac electrophysiological remodeling associated with enhanced arrhythmia susceptibility in a canine model of elite exercise
Source: eLife. 2023 Feb 23;12:e80710. doi: 10.7554/eLife.80710 (PMC10014074; doi:10.7554/eLife.80710)
Supplement: Figure 6—source data 5. [file elife-80710-fig6-data5.zip › Western blots.docx]

**A
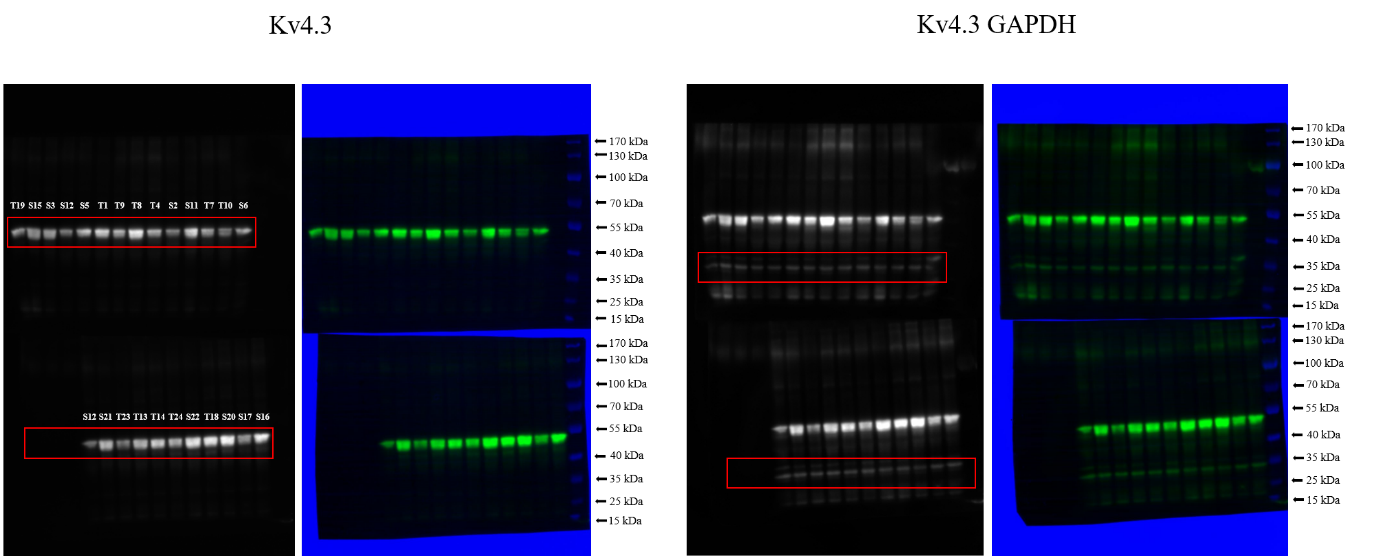
**

**B
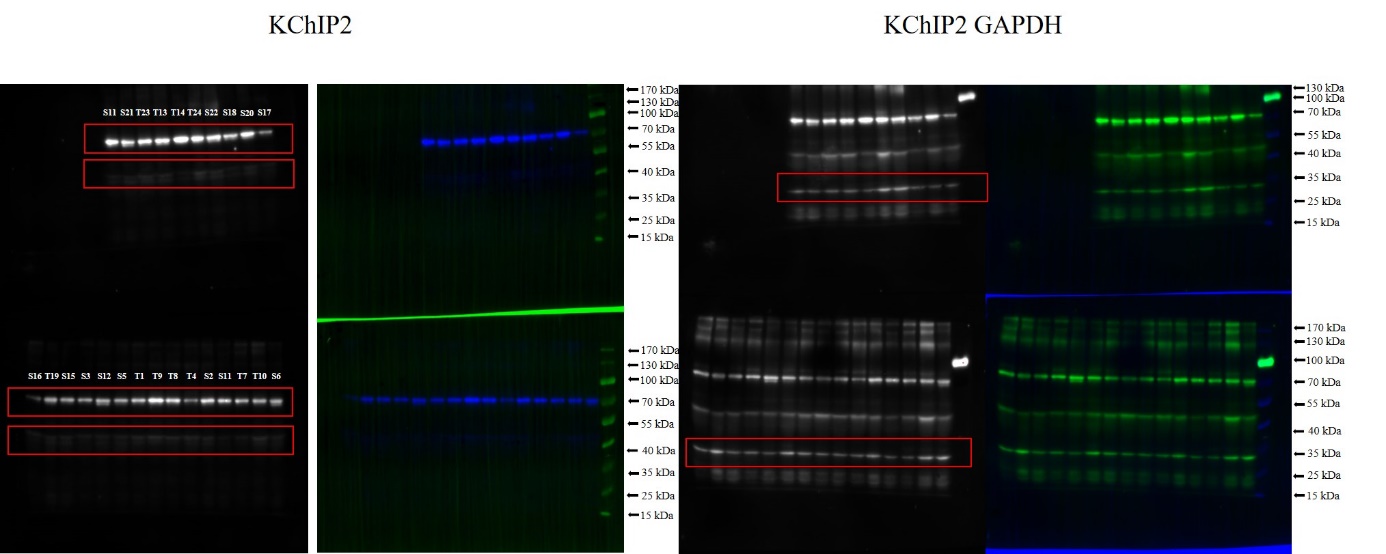
**

**Original, unedited membranes of western blots.** Kv4.3 (A) and KChIP2 (B) expression and their corresponding GAPDH controls. In the case of KChIP2 70kDa and 45kDa bands were evaluated and averaged. 70kDa bands are shown in Figure 6. Compared to the control group, training did not alter the Kv4.3 and KChIP2 expression. Kv4.3: n=12, KChIP2: n=12 animals per group. Three parallel replicates were used. T: trained group, S: sedentary group. Red boxes: cropped bands on the original films.
