# Supplementary figures and images for: Cardiac electrophysiological remodeling associated with enhanced arrhythmia susceptibility in a canine model of elite exercise
Source: eLife. 2023 Feb 23;12:e80710. doi: 10.7554/eLife.80710 (PMC10014074; doi:10.7554/eLife.80710)

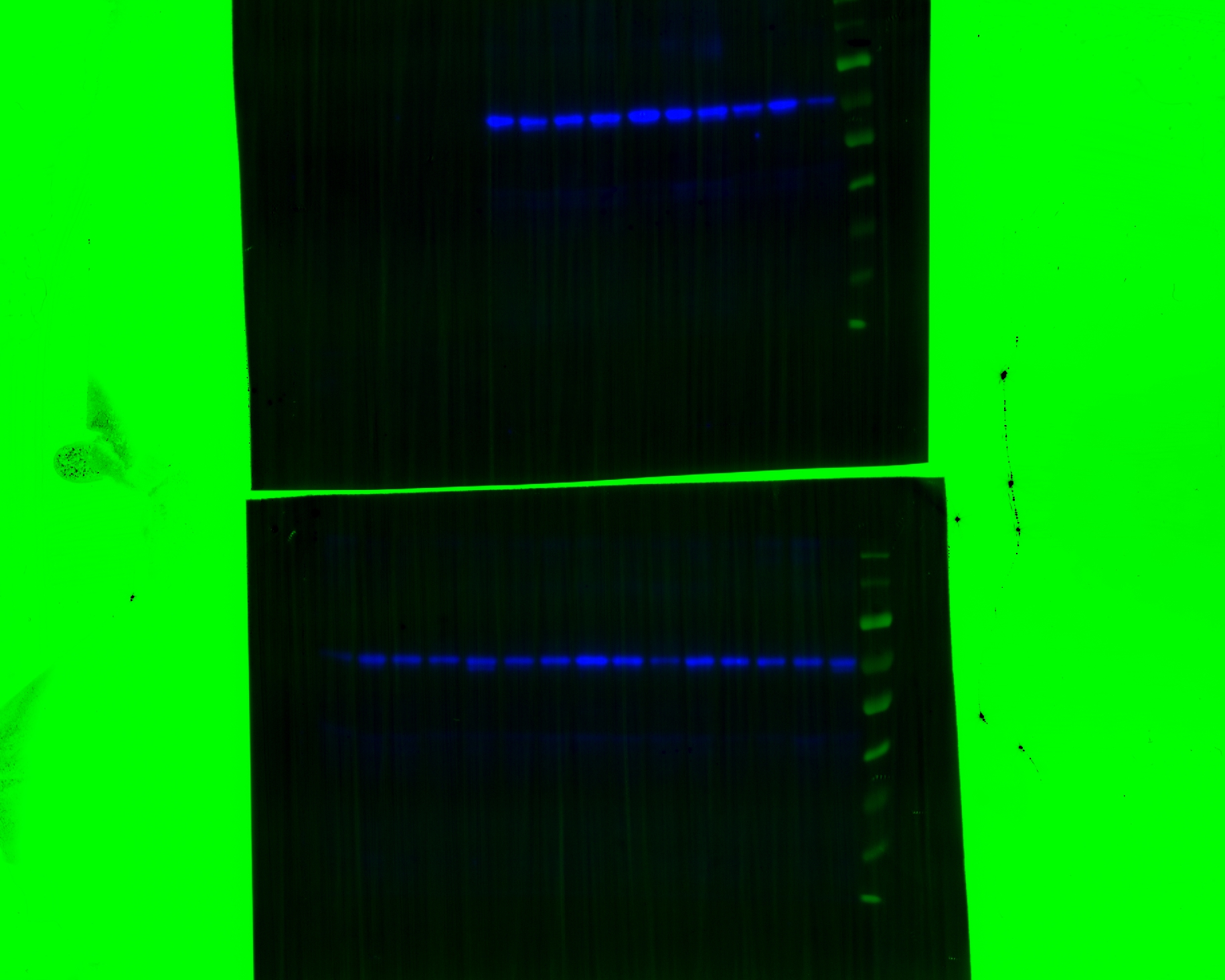

Supplement: Figure 6—source data 6. [file elife-80710-fig6-data6.zip › KChiP2 comp Episignal 2020-11-27 10h55m33s(Composite).jpg]

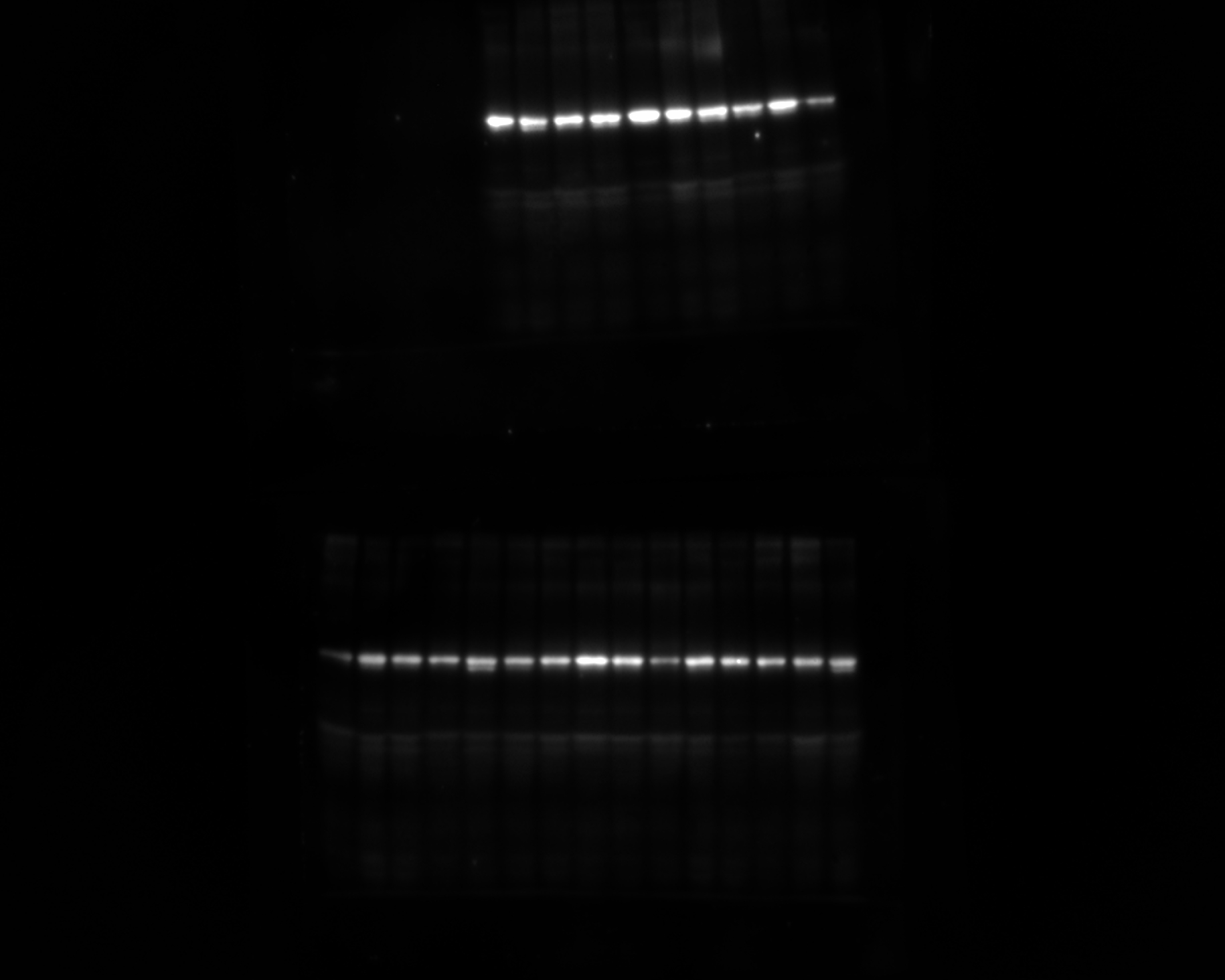

Supplement: Figure 6—source data 6. [file elife-80710-fig6-data6.zip › KChiP2 Episignal 2020-11-27 10h55m33s(Chemiluminescence).jpg]

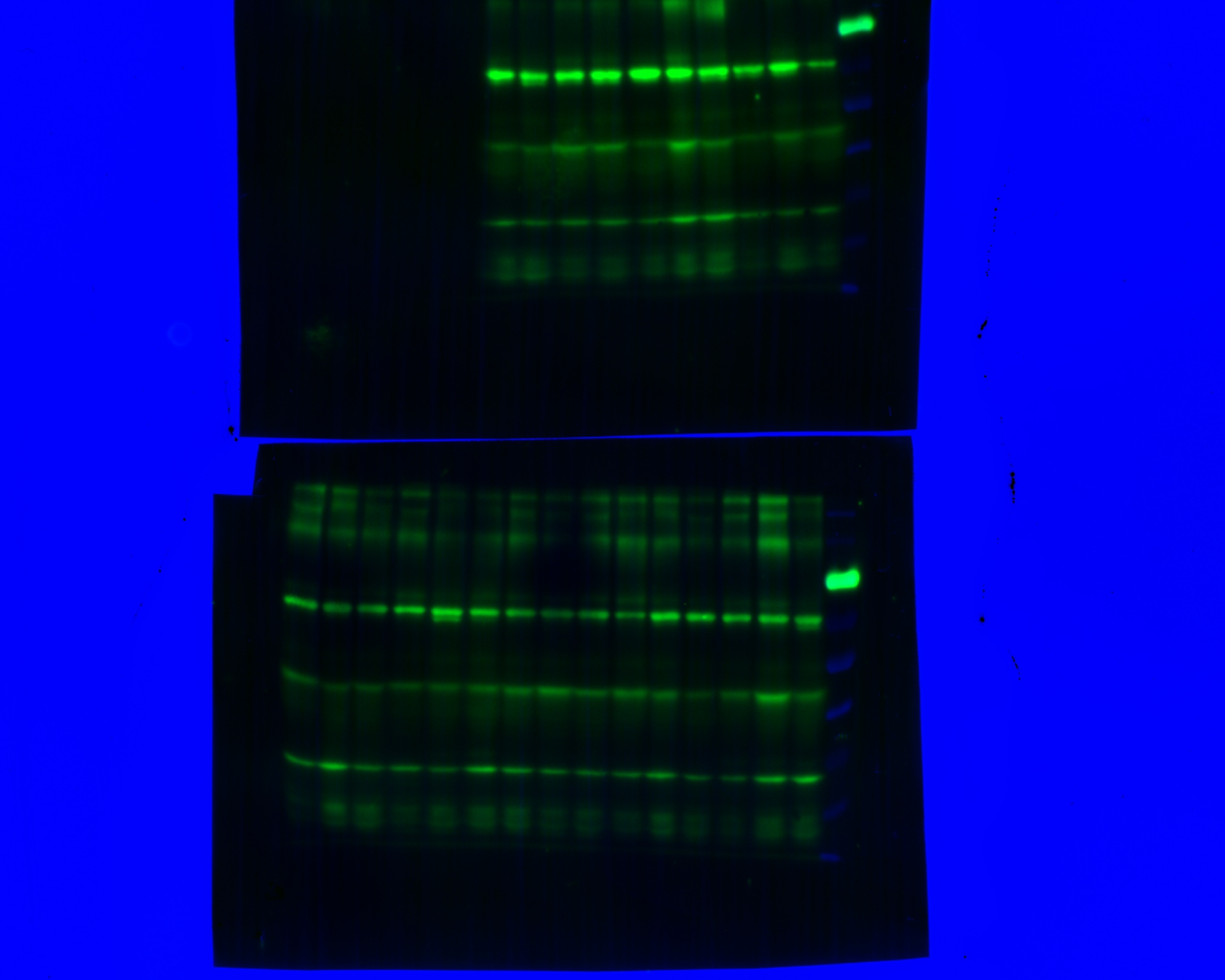

Supplement: Figure 6—source data 6. [file elife-80710-fig6-data6.zip › KChiP2 GAPDH comp Episignal 2020-12-02 11h45m44s(Composite).jpg]

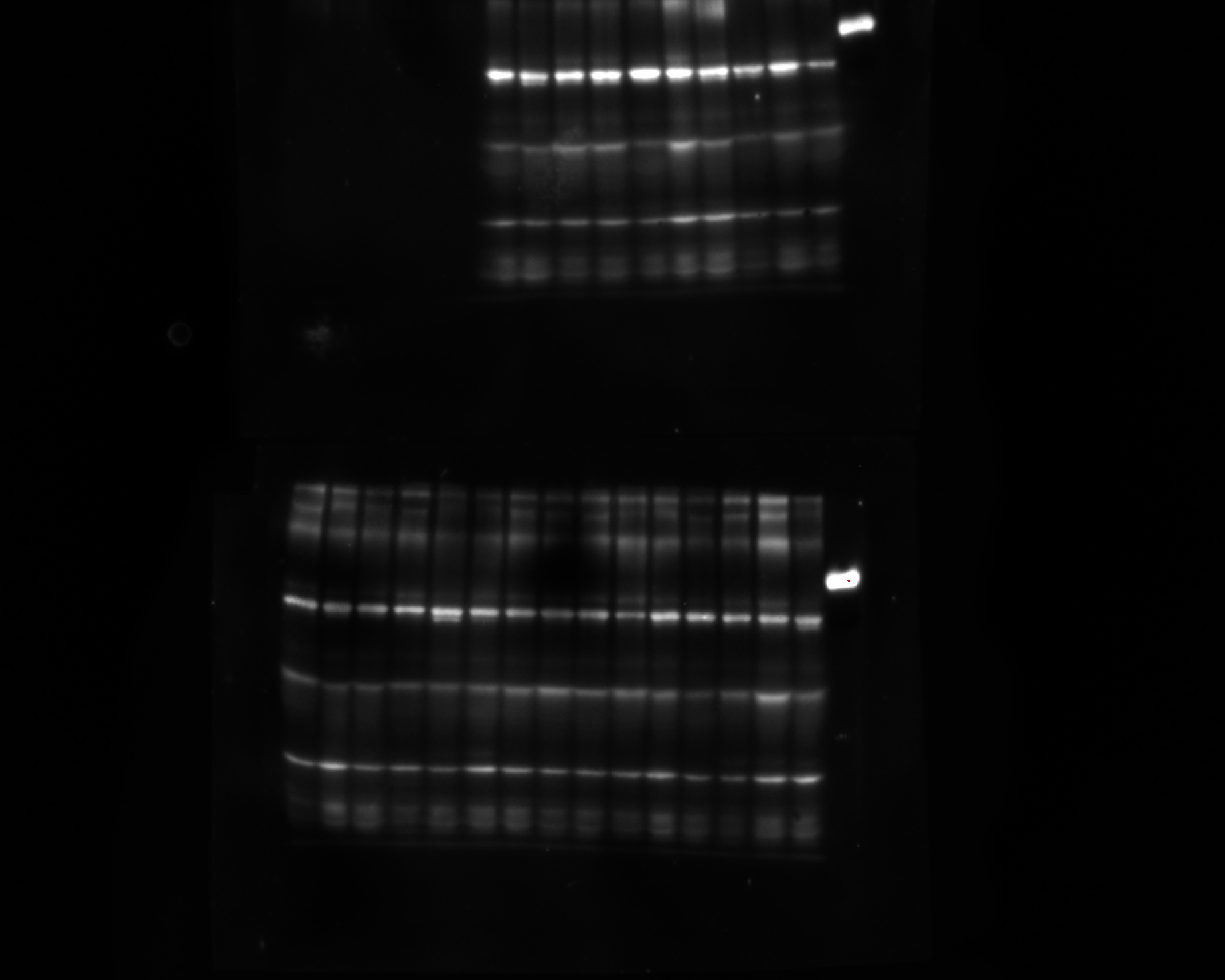

Supplement: Figure 6—source data 6. [file elife-80710-fig6-data6.zip › KChiP2 GAPDH Episignal 2020-12-02 11h45m44s(Chemiluminescence).jpg]

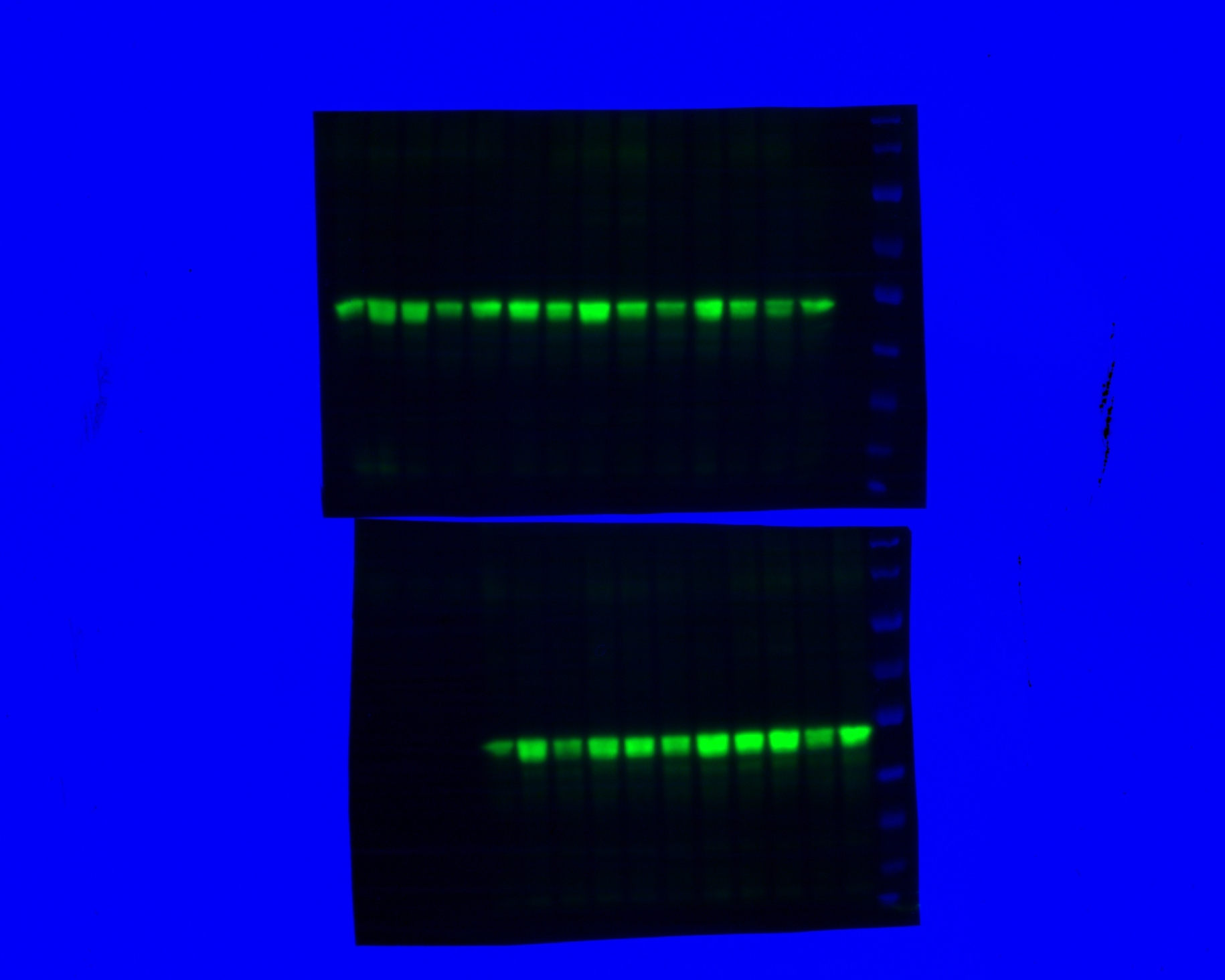

Supplement: Figure 6—source data 6. [file elife-80710-fig6-data6.zip › Kv4.3 comp Episignal 2021-03-02 12h11m09s(Composite).jpg]

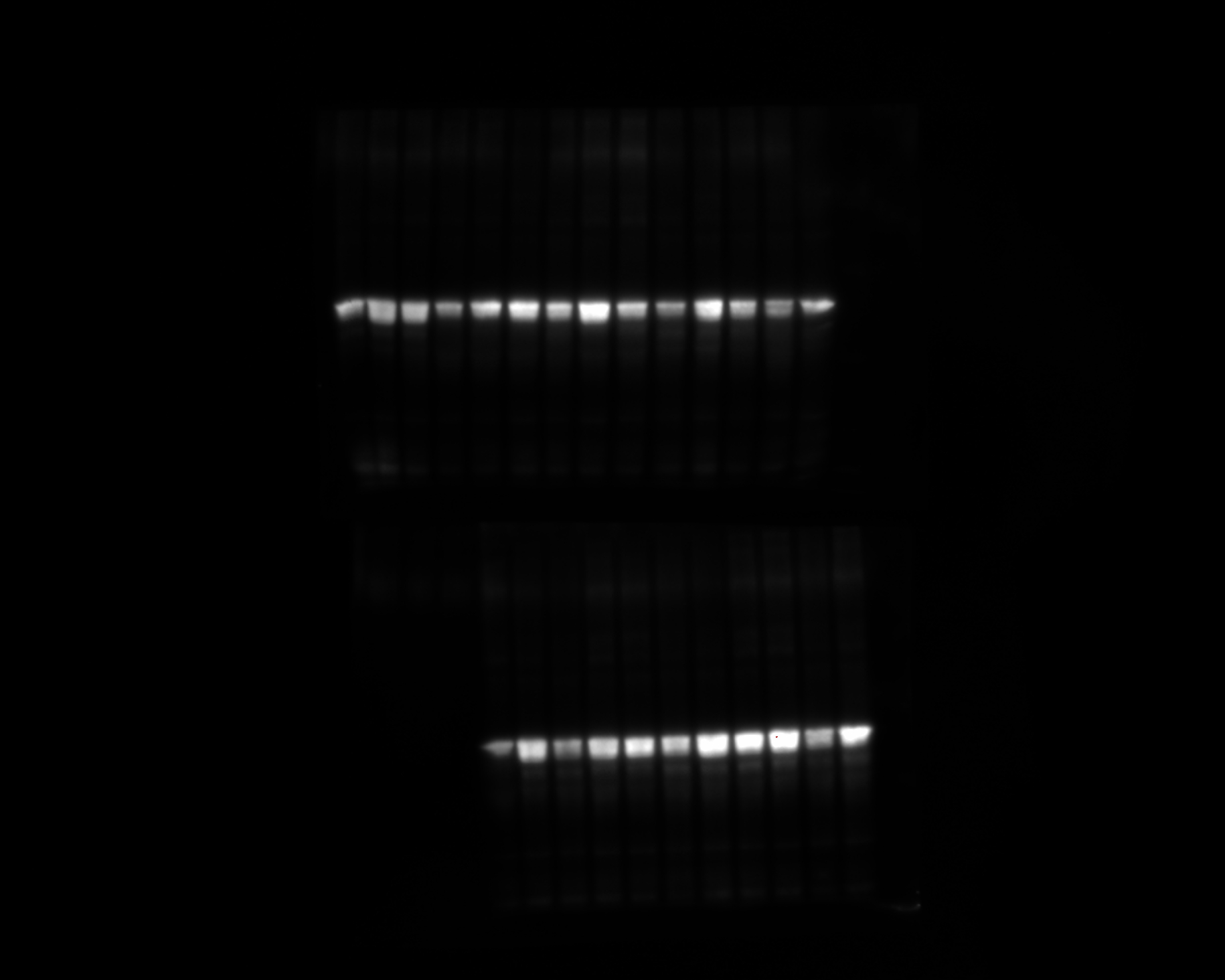

Supplement: Figure 6—source data 6. [file elife-80710-fig6-data6.zip › Kv4.3 Episignal 2021-03-02 12h11m09s(Chemiluminescence).jpg]

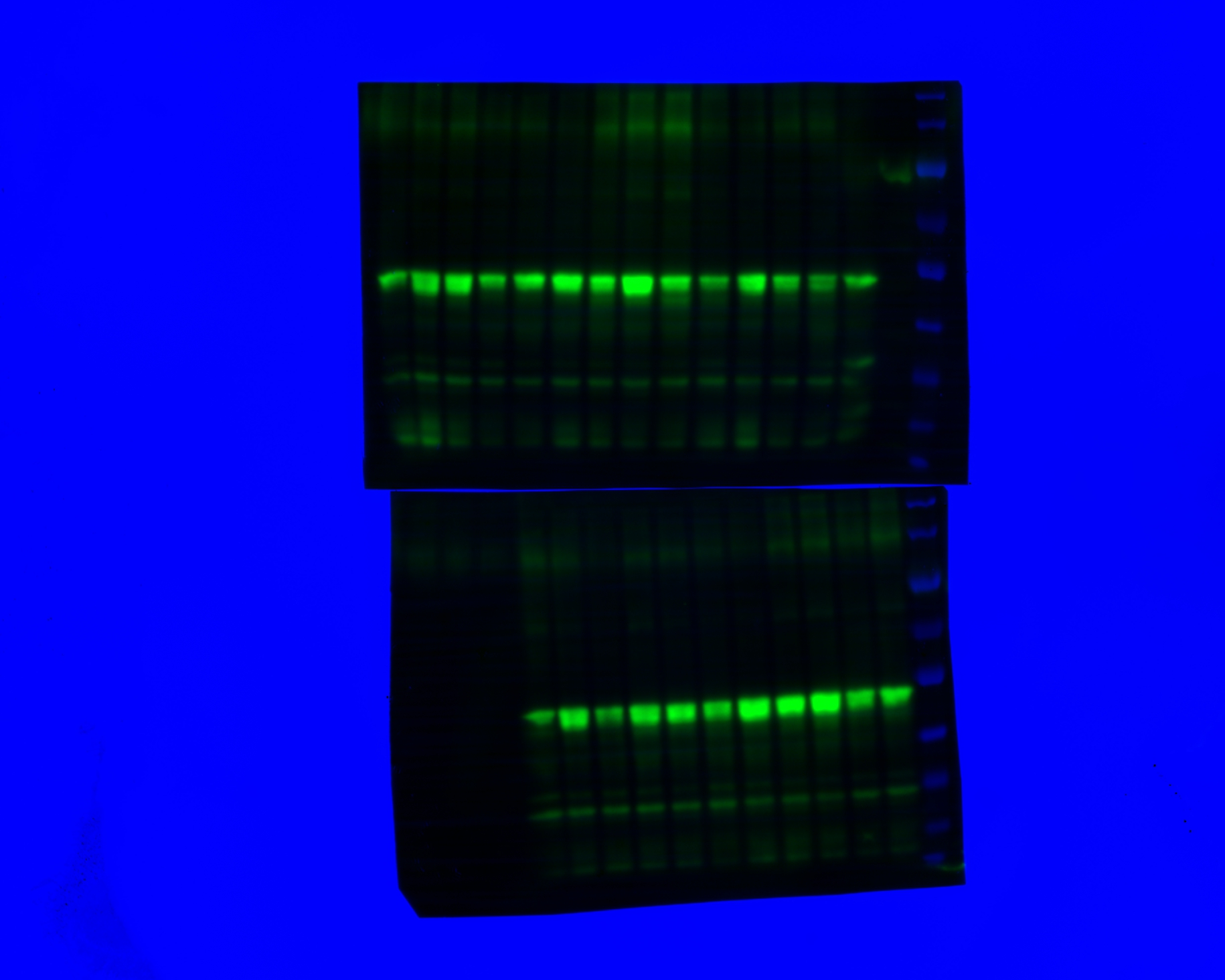

Supplement: Figure 6—source data 6. [file elife-80710-fig6-data6.zip › Kv4.3 GAPDH comp Episignal 2021-03-03 11h46m07s(Composite).jpg]

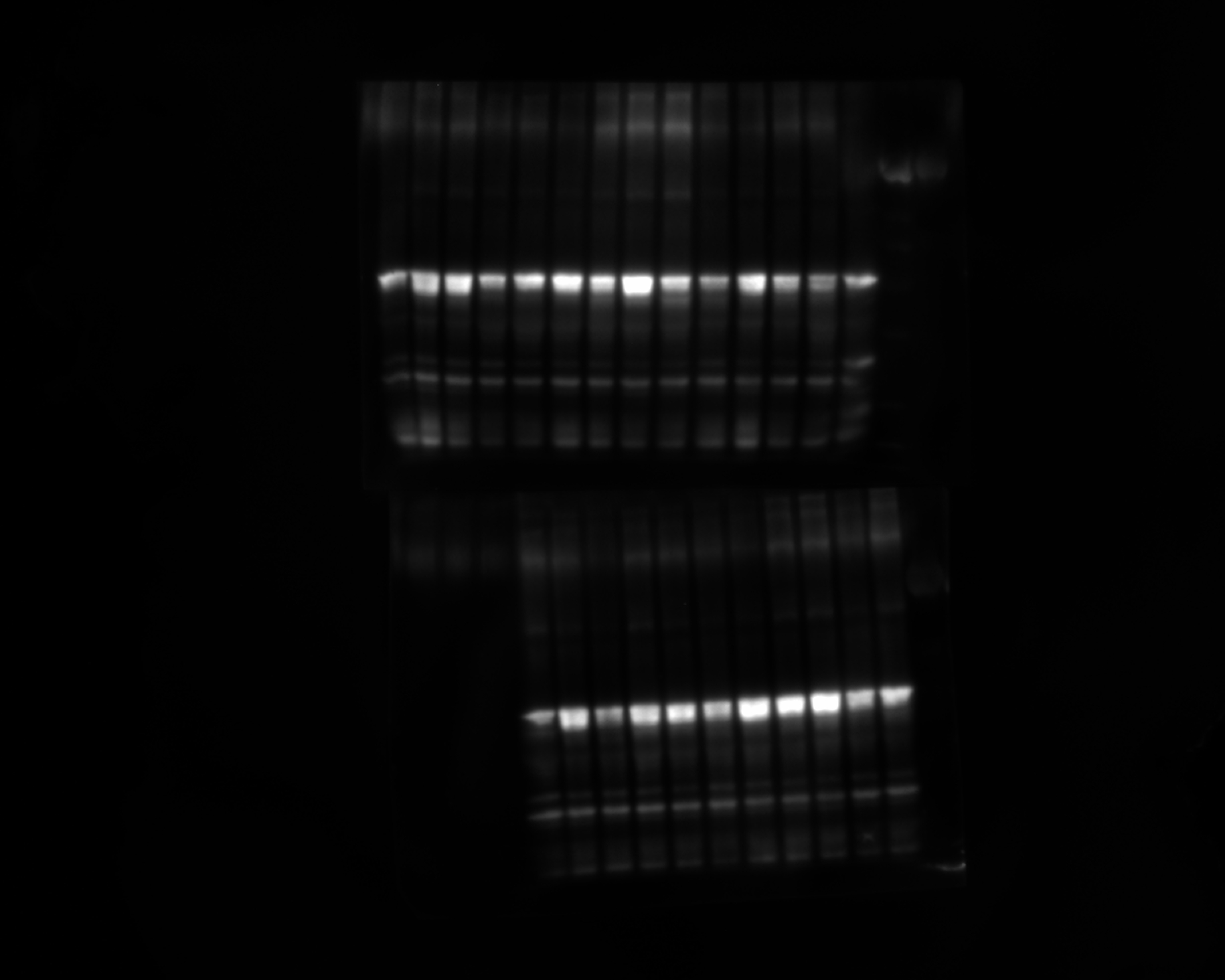

Supplement: Figure 6—source data 6. [file elife-80710-fig6-data6.zip › Kv4.3 GAPDH Episignal 2021-03-03 11h46m07s(Chemiluminescence).jpg]
